# 2-Chloro-7-methyl-12-phenyldibenzo[*b*,*g*][1,8]naphthyridin-11(6*H*)-one

**DOI:** 10.1107/S160053681002430X

**Published:** 2010-06-26

**Authors:** K. N. Vennila, K. Prabha, M. Manoj, K. J. Rajendra Prasad, D. Velmurugan

**Affiliations:** a Centre of Advanced Study in Crystallography and Biophysics, University of Madras, Guindy Campus, Chennai 600 025, India; b Department of Chemistry, Bharathiar University, Coimbatore 641 046, India

## Abstract

In the title compound, C_23_H_15_ClN_2_O, the fused ring system is planar: the deviation of all the non-H atoms from the plane through all four fused rings is less than 0.31 Å. The plane of the phenyl ring is inclined at 71.78 (5)° to the mean plane of the 1,8-naphthrydine ring system. The crystal structure is devoid of any classical hydrogen bonds but π–π inter­actions are present.

## Related literature

For the biological activity of [1,8]naphthyridine derivatives, see: Egawa *et al.* (1984 ▶); Cooper *et al.* (1992 ▶); Chen *et al.* (1997) ▶; Balin & Tan (1984 ▶); Nadaraj *et al.* (2009 ▶); Kuroda *et al.* (1992 ▶). For the synthesis of the title compound, see: Manoj *et al.* (2009 ▶). For the crystal structures of other naphthrydine derivatives, see: Sivakumar *et al.* (2003 ▶); Seebacher *et al.* (2010 ▶). For bond-length data, see: Allen *et al.* (1987 ▶). 
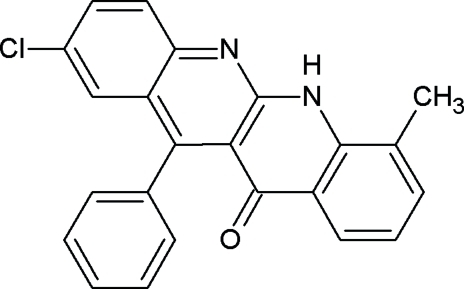


## Experimental

### 

#### Crystal data

C_23_H_15_ClN_2_O
*M* *_r_* = 370.82
Triclinic, 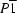

*a* = 8.2434 (2) Å
*b* = 8.5528 (2) Å
*c* = 13.0740 (3) Å
α = 89.446 (1)°
β = 74.362 (1)°
γ = 77.672 (1)°
*V* = 866.06 (4) Å^3^
*Z* = 2
Mo *K*α radiation
μ = 0.24 mm^−1^
*T* = 293 K
0.25 × 0.24 × 0.23 mm

#### Data collection

Bruker SMART APEXII area-detector diffractometer
Absorption correction: multi-scan (*SADABS*; Bruker, 2007 ▶) *T*_min_ = 0.943, *T*_max_ = 0.947
21083 measured reflections
5019 independent reflections
4047 reflections with *I* > 2σ(*I*)
*R*_int_ = 0.025

#### Refinement

*R*[*F* ^2^ > 2σ(*F* ^2^)] = 0.044
*wR*(*F* ^2^) = 0.138
*S* = 1.00
5019 reflections
245 parameters
H-atom parameters constrained
Δρ_max_ = 0.37 e Å^−3^
Δρ_min_ = −0.35 e Å^−3^

### 

Data collection: *APEX2* (Bruker, 2007 ▶); cell refinement: *SAINT* (Bruker, 2007 ▶); data reduction: *SAINT*; program(s) used to solve structure: *SHELXS97* (Sheldrick, 2008 ▶); program(s) used to refine structure: *SHELXL97* (Sheldrick, 2008 ▶); molecular graphics: *ORTEP-3* (Farrugia, 1997 ▶); software used to prepare material for publication: *SHELXL97* and *PLATON* (Spek, 2009 ▶).

## Supplementary Material

Crystal structure: contains datablocks global, I. DOI: 10.1107/S160053681002430X/su2185sup1.cif

Structure factors: contains datablocks I. DOI: 10.1107/S160053681002430X/su2185Isup2.hkl

Additional supplementary materials:  crystallographic information; 3D view; checkCIF report

## Figures and Tables

**Table 1:** π–π inter­actions (Å). *Cg*1–*Cg*4 are the centroids of the N1/C5–C9, N2/C8–C12, C1–C6 and C10—C16 rings, respectively.

*Cg*1⋯*Cg*2^i^	3.7936 (6)
*Cg*1⋯*Cg*4^ii^	3.7721 (7)
*Cg*2⋯*Cg*1^i^	3.7935 (6)
*Cg*2⋯*Cg*2^ii^	3.6542 (6)
*Cg*2⋯*Cg*3^i^	3.8725 (7)
*Cg*2⋯*Cg*4^ii^	3.5506 (7)
*Cg*3⋯*Cg*2^i^	3.8725 (7)
*Cg*3⋯*Cg*4^ii^	3.6485 (8)

Symmetry codes: (i) −*x* + 2, −*y* + 1, −*z*; (ii) −*x* + 2, −*y*, −*z*.

**Fig. 1.**
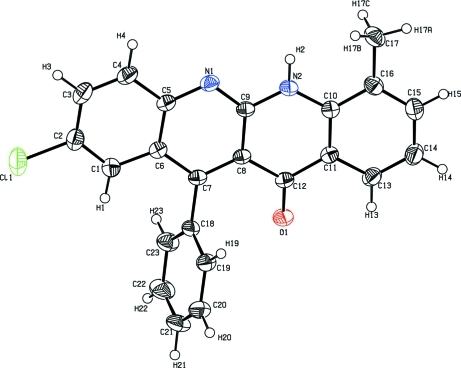
View of the title molecule, showing the thermal ellipsoids drawn at the 50% probability level.

**Fig. 2.**
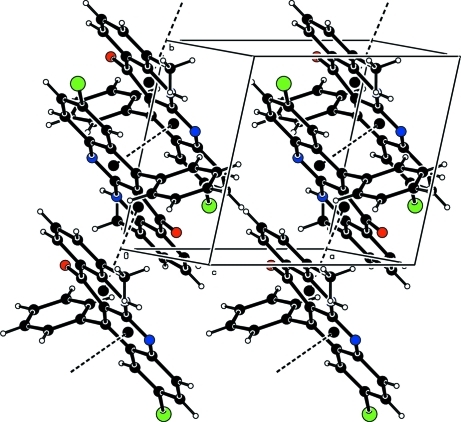
View of the crystal packing of the title compound, illustrating the π–π interactions as dotted lines [the centroids are marked by large black dots; see Table 1 for details].

## supplementary crystallographic information

### Comment

In general, nitrogen containing heterocyclic compounds play important roles in biological activities. Among such compounds, [1,8]naphthyridine derivatives represent the most active class of compounds possessing a wide spectrum of biological activities, such as antibacterial (Miyamoto *et al.*, 1984, Cooper *et al.*, 1992), antitumour (Chen *et al.*, 1997), antimalarial (Balin *et al.*, 1984), antifungal (Nadaraj *et al.*, 2009), anti-inflammatory (Kuroda *et al.*, 1992), antihypertensive activities *etc*. This paper describes the crystal structure of the title compound, which will help us in our studies on drug design.

The title compound consists of a [1,8]napthyridine core with methyl, chloro-phenyl and phenyl group substituents (Fig. 1). The dihedral angle between the two fused rings of the napthyridine moiety is found to be 3.08 (3)°, indicating that it is almost planar. The phenyl ring is inclined to the mean plane of the [1,8]naphthrydine ring system by 72.51 (3)°. The bond lengths of the formal single bonds, and C2—Cl1 = 1.7392 (14) and C12–O1 = 1.2240 (14) Å, are in normal ranges (Allen *et al.*, 1987), and similar to those observed in other naphthrydine derivatives (Sivakumar *et al.*, 2003; Seebacher *et al.*, 2010).

The crystal packing of the molecules in the crystal is influenced by π–π interactions and van der Waals forces (Fig. 2 and Table 1).

Footnote for Table 1: Symmetry codes : (i) -x+2, -y+1, -z; (ii) -x+2, -y, -z.

### Experimental

The title compund was synthesized according to the published procedure (Manoj *et al.*, 2009). 2[(2'-Benzoyl-4'-chlorophenyl)amino]-4-chloroquinoline (2 mmol) was added to polyphosphoric acid (6 g of P_2_O_5_ in 3 ml of H_3_PO_4_) and heated at 478–483 K for 5 h. The reaction was monitored by using TLC. After completion of the reaction, the reaction mixture was poured into ice water and extracted with ethyl acetate. It was then purified by column chromatography using silica gel and the product eluted with a petroleum ether:ethyl acetate (96:4) mixture, to give the title compound as a pale yellow solid. It was recrystallized using methanol.

### Refinement

The H-atoms were positioned geometrically and treated as riding atoms: C—H = 0.93 Å H-aromatic, C—H = 0.96 Å H-methyl, and N—H = 0.86 Å, with *U*_iso_ = k×*U*_eq_(parent C or N-atom), where k = 1.5 for methyl H-atoms, and = 1.2 for all other H-atoms.

### Crystal data

**Table:**

C_23_H_15_ClN_2_O	*Z* = 2
*M**_r_* = 370.82	*F*(000) = 384
Triclinic, *P*1	*D*_x_ = 1.422 Mg m^−^^3^
Hall symbol: -P 1	Mo *K*α radiation, λ = 0.71073 Å
*a* = 8.2434 (2) Å	Cell parameters from 5019 reflections
*b* = 8.5528 (2) Å	θ = 1.6–30.0°
*c* = 13.0740 (3) Å	µ = 0.24 mm^−^^1^
α = 89.446 (1)°	*T* = 293 K
β = 74.362 (1)°	Block, pale yellow
γ = 77.672 (1)°	0.25 × 0.24 × 0.23 mm
*V* = 866.06 (4) Å^3^	

### Data collection

**Table:**

Bruker SMART APEXII area-detector diffractometer	5019 independent reflections
Radiation source: fine-focus sealed tube	4047 reflections with *I* > 2σ(*I*)
graphite	*R*_int_ = 0.025
ω and φ scans	θ_max_ = 30.0°, θ_min_ = 2.4°
Absorption correction: multi-scan (*SADABS*; Bruker, 2007)	*h* = −11→11
*T*_min_ = 0.943, *T*_max_ = 0.947	*k* = −12→12
21083 measured reflections	*l* = −17→18

### Refinement

**Table:**

Refinement on *F*^2^	Primary atom site location: structure-invariant direct methods
Least-squares matrix: full	Secondary atom site location: difference Fourier map
*R*[*F*^2^ > 2σ(*F*^2^)] = 0.044	Hydrogen site location: inferred from neighbouring sites
*wR*(*F*^2^) = 0.138	H-atom parameters constrained
*S* = 1.00	*w* = 1/[σ^2^(*F*_o_^2^) + (0.0812*P*)^2^ + 0.1735*P*] where *P* = (*F*_o_^2^ + 2*F*_c_^2^)/3
5019 reflections	(Δ/σ)_max_ < 0.001
245 parameters	Δρ_max_ = 0.37 e Å^−^^3^
0 restraints	Δρ_min_ = −0.34 e Å^−^^3^

### Special details

**Table:**

Geometry. All esds (except the esd in the dihedral angle between two l.s. planes) are estimated using the full covariance matrix. The cell esds are taken into account individually in the estimation of esds in distances, angles and torsion angles; correlations between esds in cell parameters are only used when they are defined by crystal symmetry. An approximate (isotropic) treatment of cell esds is used for estimating esds involving l.s. planes.
Refinement. Refinement of F^2^ against ALL reflections. The weighted R-factor wR and goodness of fit S are based on F^2^, conventional R-factors R are based on F, with F set to zero for negative F^2^. The threshold expression of F^2^ > 2sigma(F^2^) is used only for calculating R-factors(gt) etc. and is not relevant to the choice of reflections for refinement. R-factors based on F^2^ are statistically about twice as large as those based on F, and R- factors based on ALL data will be even larger.

### Fractional atomic coordinates and isotropic or equivalent isotropic displacement parameters (Å^2^)

**Table:**

	*x*	*y*	*z*	*U*_iso_*/*U*_eq_	
Cl1	0.43107 (6)	0.82442 (6)	0.41605 (3)	0.06692 (16)	
C8	0.97978 (14)	0.28540 (13)	0.08976 (9)	0.0295 (2)	
N1	0.75659 (13)	0.43848 (12)	0.01596 (8)	0.0347 (2)	
C6	0.75767 (14)	0.49589 (13)	0.19809 (9)	0.0321 (2)	
C7	0.91173 (14)	0.37615 (13)	0.18528 (9)	0.0303 (2)	
C11	1.19508 (14)	0.07433 (13)	−0.03719 (9)	0.0318 (2)	
N2	0.96411 (13)	0.24442 (12)	−0.08970 (8)	0.0349 (2)	
H2	0.9104	0.2694	−0.1376	0.042*	
C5	0.68564 (14)	0.52218 (14)	0.11069 (9)	0.0329 (2)	
C9	0.89694 (14)	0.32538 (13)	0.00739 (9)	0.0298 (2)	
O1	1.18861 (13)	0.08963 (12)	0.14332 (7)	0.0456 (2)	
C16	1.17758 (17)	0.05651 (15)	−0.22018 (10)	0.0375 (3)	
C12	1.12754 (15)	0.14578 (14)	0.07187 (9)	0.0318 (2)	
C10	1.11201 (14)	0.12564 (13)	−0.11549 (9)	0.0317 (2)	
C15	1.32690 (18)	−0.06129 (16)	−0.24169 (11)	0.0443 (3)	
H15	1.3730	−0.1075	−0.3103	0.053*	
C19	1.15161 (16)	0.40958 (15)	0.25824 (10)	0.0390 (3)	
H19	1.1985	0.4551	0.1953	0.047*	
C18	0.99944 (15)	0.35745 (14)	0.27169 (9)	0.0334 (2)	
C13	1.34488 (16)	−0.04709 (15)	−0.06305 (11)	0.0392 (3)	
H13	1.3998	−0.0829	−0.0110	0.047*	
C1	0.67473 (16)	0.59156 (15)	0.29368 (10)	0.0388 (3)	
H1	0.7190	0.5754	0.3522	0.047*	
C4	0.53475 (16)	0.64433 (16)	0.12077 (12)	0.0423 (3)	
H4	0.4868	0.6619	0.0638	0.051*	
C23	0.92949 (19)	0.29264 (18)	0.36673 (11)	0.0451 (3)	
H23	0.8252	0.2611	0.3775	0.054*	
C17	1.0871 (2)	0.11093 (18)	−0.30353 (10)	0.0472 (3)	
H17A	1.1601	0.0680	−0.3721	0.071*	
H17B	1.0616	0.2259	−0.3030	0.071*	
H17C	0.9815	0.0738	−0.2890	0.071*	
C2	0.53032 (17)	0.70682 (16)	0.29904 (11)	0.0424 (3)	
C20	1.23394 (19)	0.39418 (18)	0.33819 (12)	0.0494 (3)	
H20	1.3351	0.4308	0.3293	0.059*	
C21	1.1663 (2)	0.3246 (2)	0.43107 (12)	0.0584 (4)	
H21	1.2237	0.3114	0.4839	0.070*	
C14	1.41133 (18)	−0.11368 (17)	−0.16453 (12)	0.0465 (3)	
H14	1.5121	−0.1933	−0.1818	0.056*	
C22	1.0139 (2)	0.2747 (2)	0.44566 (12)	0.0584 (4)	
H22	0.9678	0.2288	0.5086	0.070*	
C3	0.45924 (17)	0.73618 (17)	0.21266 (13)	0.0468 (3)	
H3	0.3618	0.8174	0.2181	0.056*	

### Atomic displacement parameters (Å^2^)

**Table:**

	*U*^11^	*U*^22^	*U*^33^	*U*^12^	*U*^13^	*U*^23^
Cl1	0.0572 (2)	0.0680 (3)	0.0571 (2)	0.00139 (19)	0.00421 (18)	−0.01642 (19)
C8	0.0295 (5)	0.0310 (5)	0.0304 (5)	−0.0063 (4)	−0.0127 (4)	0.0058 (4)
N1	0.0342 (5)	0.0353 (5)	0.0368 (5)	−0.0039 (4)	−0.0166 (4)	0.0056 (4)
C6	0.0303 (5)	0.0321 (5)	0.0349 (5)	−0.0068 (4)	−0.0106 (4)	0.0034 (4)
C7	0.0309 (5)	0.0330 (5)	0.0305 (5)	−0.0087 (4)	−0.0130 (4)	0.0063 (4)
C11	0.0307 (5)	0.0314 (5)	0.0345 (5)	−0.0076 (4)	−0.0103 (4)	0.0035 (4)
N2	0.0372 (5)	0.0389 (5)	0.0299 (5)	−0.0033 (4)	−0.0155 (4)	0.0034 (4)
C5	0.0309 (5)	0.0322 (5)	0.0382 (6)	−0.0067 (4)	−0.0140 (4)	0.0053 (4)
C9	0.0318 (5)	0.0313 (5)	0.0298 (5)	−0.0080 (4)	−0.0133 (4)	0.0066 (4)
O1	0.0491 (5)	0.0473 (5)	0.0404 (5)	0.0056 (4)	−0.0247 (4)	0.0027 (4)
C16	0.0418 (6)	0.0399 (6)	0.0325 (6)	−0.0139 (5)	−0.0091 (5)	0.0031 (4)
C12	0.0315 (5)	0.0326 (5)	0.0340 (5)	−0.0059 (4)	−0.0143 (4)	0.0053 (4)
C10	0.0320 (5)	0.0317 (5)	0.0324 (5)	−0.0092 (4)	−0.0091 (4)	0.0041 (4)
C15	0.0437 (7)	0.0443 (7)	0.0405 (6)	−0.0096 (5)	−0.0038 (5)	−0.0056 (5)
C19	0.0381 (6)	0.0406 (6)	0.0405 (6)	−0.0065 (5)	−0.0159 (5)	0.0020 (5)
C18	0.0359 (5)	0.0350 (5)	0.0305 (5)	−0.0035 (4)	−0.0144 (4)	0.0003 (4)
C13	0.0341 (6)	0.0363 (6)	0.0474 (7)	−0.0034 (4)	−0.0147 (5)	0.0014 (5)
C1	0.0369 (6)	0.0410 (6)	0.0377 (6)	−0.0077 (5)	−0.0092 (5)	−0.0008 (5)
C4	0.0352 (6)	0.0403 (6)	0.0525 (7)	−0.0018 (5)	−0.0190 (5)	0.0055 (5)
C23	0.0489 (7)	0.0552 (8)	0.0357 (6)	−0.0156 (6)	−0.0161 (5)	0.0082 (5)
C17	0.0581 (8)	0.0523 (7)	0.0315 (6)	−0.0100 (6)	−0.0144 (6)	0.0013 (5)
C2	0.0353 (6)	0.0403 (6)	0.0466 (7)	−0.0070 (5)	−0.0033 (5)	−0.0034 (5)
C20	0.0447 (7)	0.0542 (8)	0.0553 (8)	−0.0081 (6)	−0.0259 (6)	−0.0069 (6)
C21	0.0677 (10)	0.0708 (10)	0.0445 (8)	−0.0060 (8)	−0.0353 (7)	−0.0027 (7)
C14	0.0369 (6)	0.0438 (7)	0.0534 (8)	−0.0012 (5)	−0.0088 (6)	−0.0056 (6)
C22	0.0709 (10)	0.0748 (11)	0.0352 (7)	−0.0160 (8)	−0.0242 (7)	0.0126 (7)
C3	0.0319 (6)	0.0414 (7)	0.0624 (9)	−0.0008 (5)	−0.0106 (6)	0.0006 (6)

### Geometric parameters (Å, °)

**Table:**

Cl1—C2	1.7392 (14)	C19—C20	1.3828 (18)
C8—C7	1.3899 (15)	C19—C18	1.3863 (17)
C8—C9	1.4256 (14)	C19—H19	0.9300
C8—C12	1.4816 (15)	C18—C23	1.3859 (18)
N1—C9	1.3196 (14)	C13—C14	1.3690 (19)
N1—C5	1.3551 (15)	C13—H13	0.9300
C6—C5	1.4187 (16)	C1—C2	1.3598 (18)
C6—C1	1.4210 (16)	C1—H1	0.9300
C6—C7	1.4223 (15)	C4—C3	1.362 (2)
C7—C18	1.4873 (15)	C4—H4	0.9300
C11—C10	1.3965 (16)	C23—C22	1.3824 (19)
C11—C13	1.3971 (16)	C23—H23	0.9300
C11—C12	1.4697 (16)	C17—H17A	0.9600
N2—C9	1.3681 (15)	C17—H17B	0.9600
N2—C10	1.3741 (15)	C17—H17C	0.9600
N2—H2	0.8600	C2—C3	1.404 (2)
C5—C4	1.4197 (16)	C20—C21	1.379 (2)
O1—C12	1.2240 (14)	C20—H20	0.9300
C16—C15	1.3780 (18)	C21—C22	1.376 (2)
C16—C10	1.4126 (16)	C21—H21	0.9300
C16—C17	1.4970 (18)	C14—H14	0.9300
C15—C14	1.393 (2)	C22—H22	0.9300
C15—H15	0.9300	C3—H3	0.9300
			
C7—C8—C9	117.71 (10)	C23—C18—C7	121.22 (11)
C7—C8—C12	123.22 (9)	C19—C18—C7	119.52 (10)
C9—C8—C12	119.00 (10)	C14—C13—C11	120.42 (12)
C9—N1—C5	117.25 (10)	C14—C13—H13	119.8
C5—C6—C1	118.78 (10)	C11—C13—H13	119.8
C5—C6—C7	118.27 (10)	C2—C1—C6	119.61 (12)
C1—C6—C7	122.95 (10)	C2—C1—H1	120.2
C8—C7—C6	118.53 (10)	C6—C1—H1	120.2
C8—C7—C18	122.12 (10)	C3—C4—C5	120.95 (12)
C6—C7—C18	119.28 (10)	C3—C4—H4	119.5
C10—C11—C13	119.38 (11)	C5—C4—H4	119.5
C10—C11—C12	121.14 (10)	C22—C23—C18	120.35 (13)
C13—C11—C12	119.48 (11)	C22—C23—H23	119.8
C9—N2—C10	124.08 (10)	C18—C23—H23	119.8
C9—N2—H2	118.0	C16—C17—H17A	109.5
C10—N2—H2	118.0	C16—C17—H17B	109.5
N1—C5—C6	123.07 (10)	H17A—C17—H17B	109.5
N1—C5—C4	117.74 (11)	C16—C17—H17C	109.5
C6—C5—C4	119.16 (11)	H17A—C17—H17C	109.5
N1—C9—N2	115.15 (10)	H17B—C17—H17C	109.5
N1—C9—C8	125.05 (10)	C1—C2—C3	122.28 (12)
N2—C9—C8	119.80 (10)	C1—C2—Cl1	119.20 (11)
C15—C16—C10	117.34 (11)	C3—C2—Cl1	118.51 (10)
C15—C16—C17	122.02 (12)	C21—C20—C19	120.08 (13)
C10—C16—C17	120.64 (11)	C21—C20—H20	120.0
O1—C12—C11	121.25 (11)	C19—C20—H20	120.0
O1—C12—C8	122.72 (11)	C22—C21—C20	120.06 (13)
C11—C12—C8	116.02 (9)	C22—C21—H21	120.0
N2—C10—C11	119.27 (10)	C20—C21—H21	120.0
N2—C10—C16	119.86 (11)	C13—C14—C15	119.50 (12)
C11—C10—C16	120.87 (11)	C13—C14—H14	120.3
C16—C15—C14	122.47 (12)	C15—C14—H14	120.3
C16—C15—H15	118.8	C21—C22—C23	120.02 (14)
C14—C15—H15	118.8	C21—C22—H22	120.0
C20—C19—C18	120.20 (13)	C23—C22—H22	120.0
C20—C19—H19	119.9	C4—C3—C2	119.20 (12)
C18—C19—H19	119.9	C4—C3—H3	120.4
C23—C18—C19	119.24 (11)	C2—C3—H3	120.4
			
C9—C8—C7—C6	4.14 (15)	C13—C11—C10—C16	−0.06 (17)
C12—C8—C7—C6	−172.83 (10)	C12—C11—C10—C16	179.99 (10)
C9—C8—C7—C18	−172.72 (10)	C15—C16—C10—N2	179.56 (11)
C12—C8—C7—C18	10.31 (17)	C17—C16—C10—N2	−0.24 (17)
C5—C6—C7—C8	−3.06 (16)	C15—C16—C10—C11	−0.77 (17)
C1—C6—C7—C8	177.96 (10)	C17—C16—C10—C11	179.43 (11)
C5—C6—C7—C18	173.90 (10)	C10—C16—C15—C14	0.73 (19)
C1—C6—C7—C18	−5.09 (17)	C17—C16—C15—C14	−179.47 (13)
C9—N1—C5—C6	1.61 (17)	C20—C19—C18—C23	1.18 (19)
C9—N1—C5—C4	179.91 (10)	C20—C19—C18—C7	179.49 (12)
C1—C6—C5—N1	179.14 (11)	C8—C7—C18—C23	−111.25 (14)
C7—C6—C5—N1	0.11 (17)	C6—C7—C18—C23	71.91 (15)
C1—C6—C5—C4	0.86 (17)	C8—C7—C18—C19	70.48 (15)
C7—C6—C5—C4	−178.16 (10)	C6—C7—C18—C19	−106.37 (13)
C5—N1—C9—N2	−179.86 (10)	C10—C11—C13—C14	0.97 (19)
C5—N1—C9—C8	−0.39 (17)	C12—C11—C13—C14	−179.07 (11)
C10—N2—C9—N1	178.18 (10)	C5—C6—C1—C2	−0.80 (18)
C10—N2—C9—C8	−1.32 (17)	C7—C6—C1—C2	178.18 (11)
C7—C8—C9—N1	−2.55 (17)	N1—C5—C4—C3	−178.17 (12)
C12—C8—C9—N1	174.55 (10)	C6—C5—C4—C3	0.19 (19)
C7—C8—C9—N2	176.90 (10)	C19—C18—C23—C22	−2.4 (2)
C12—C8—C9—N2	−6.00 (16)	C7—C18—C23—C22	179.30 (13)
C10—C11—C12—O1	171.91 (11)	C6—C1—C2—C3	−0.3 (2)
C13—C11—C12—O1	−8.05 (17)	C6—C1—C2—Cl1	−179.41 (9)
C10—C11—C12—C8	−6.49 (16)	C18—C19—C20—C21	1.0 (2)
C13—C11—C12—C8	173.55 (10)	C19—C20—C21—C22	−1.9 (2)
C7—C8—C12—O1	8.09 (18)	C11—C13—C14—C15	−1.0 (2)
C9—C8—C12—O1	−168.84 (11)	C16—C15—C14—C13	0.2 (2)
C7—C8—C12—C11	−173.54 (10)	C20—C21—C22—C23	0.7 (3)
C9—C8—C12—C11	9.53 (15)	C18—C23—C22—C21	1.5 (2)
C9—N2—C10—C11	4.61 (17)	C5—C4—C3—C2	−1.3 (2)
C9—N2—C10—C16	−175.71 (10)	C1—C2—C3—C4	1.4 (2)
C13—C11—C10—N2	179.62 (11)	Cl1—C2—C3—C4	−179.52 (11)
C12—C11—C10—N2	−0.34 (17)		

### Table 1 π–π interactions (Å). Cg1–Cg4 are the centroids of rings N1/C5–C9, N2/C8–C12, C1–C6 and C10-C16, respectively.

Cg1–Cg4 are the centroids of the N1/C5–C9, N2/C8–C12, C1–C6 and C10-C16 rings, respectively.

**Table:**

Cg1···Cg2^i^	3.7936 (6)
Cg1···Cg4^ii^	3.7721 (7)
Cg2···Cg1^i^	3.7935 (6)
Cg2···Cg2^ii^	3.6542 (6)
Cg2···Cg3^i^	3.8725 (7)
Cg2···Cg4^ii^	3.5506 (7)
Cg3···Cg2^i^	3.8725 (7)
Cg3···Cg4^ii^	3.6485 (8)

Symmetry codes: (i) -x+2, -y+1, -z; (ii) -x+2, -y, -z.
